# An Essay on the Opium Trade, Including a Sketch of Its History, Extent, Effects, Etc., as Carried on in India and China

**Published:** 1850-06

**Authors:** 


*An Essay on the Opium Trade, including a Sketch of its History, Extent, Effects, etc., as carried on in India and China.* By Nathan Allen, M. D. Boston. John P. Jewett & Co. 1850. pp. 68.

The author of the above pamphlet has presented us with a fund of useful and interesting material that we truly wish we could place in the hands of all our readers. Want of space, however, compels us to be satisfied with merely culling from it some details that cannot fail to be attractive to the medical inquirer, at the same time that we commend the whole to them for careful perusal.

The extent to which the cultivation of the poppy and the trade in opium are carried on in India is almost incredible. In speaking of the cultivation of this plant, the author of this essay states that " it is estimated that more than 100,000 acres of the rich plains of central India, as well as the alluvial valley of the Ganges, are now occupied for this purpose. Formerly these same grounds were used for the production of sugar, indigo, corn and other grain, but these useful crops have yielded to the more profitable culture of the poppy." The greater part of the opium manufactured in India is consumed by the Chinese, not for medical purposes, but as a luxury. " The principal use made of opium by the Chinese is in the form of smoking, and one great object in the trade is to furnish an article adapted to their peculiar tastes. This depends somewhat upon the cultivation of the poppy, the quality of its seed, the goodness of the soil, the manner of collecting and converting its juice into a dry extract or balls convenient for transportation. The Chinese value any sample of opium in direct proportion to the quantity of hot drawn, watery extract obtainable from it, and to the purity and strength of that extract when dried and smoked through a pipe." To improve its flavor and increase its strength, the opium, after its arrival in China, is subjected to various processes. Old smokers will retain the smoke a long time in the lungs, allowing it finally to escape through the nose. As the drug is expensive, those who are poor study to use it so as to obtain the greatest effect possible from the smallest quantity.

It is supposed that between four and five millions of persons in China are addicted to the vice of opium smoking, and that on an average each person consumes upwards of seventeen grains per day. The quantity consumed depends very much on the habits of the smoker; at first he cannot inhale more than from three to six grains at a time, but will go on gradually increasing the quantity, till in a few years his daily allowance will reach three hundred grains. It is calculated that the victims of this vice do not live on an average more than *ten years* after they have once fairly given way to the habit. According to this calculation, between four and five hundred thousand persons die annually in China from the deleterious effects of opium smoking. Well might the *native* remark: *It is not the man who eats opium, but it is opium that eats the man.*

"The practice of *eating* opium, as a luxury, has prevailed for more than a century in Persia and Turkey, but that of *smoking* it originated at a much later period, and has been confined mostly to China and its adjacent provinces. The effects of the latter practice, we believe, are far more pernicious than the former." A distinguished Chinese scholar in a memorial to the Emperor says: "Opium is a poisonous drug, brought from foreign countries, and, when the poison takes effect, the habit becomes fixed, and the sleeping smokers are like corpses—lean and haggard as demons." So strongly does the vice of opium-smoking fasten itself upon its victim, that having fairly contracted the habit at twenty, he may expect to die at the age of thirty years. It is very rarely the case that an habitual opium-smoker relinquishes the practice till his wretched career is ended by death.

Of the destructive effects of this drug upon an entire community, the island of Formosa, situated in the Chinese seas, and the province of Assam, lying on the eastern frontier of Bengal, afford most striking examples. Of the latter it is said, since the introduction of opium, "the women have fewer children compared with those of other countries, and the children seldom live to become old men, but in general die at early manhood; very few old men being seen in this unfortunate country, compared with others." In regard to the former the following remarks occur:—"The natives of this place were at first sprightly and active, and being good soldiers were always successful in battle, but the people called Hung-maou came thither, and having manufactured opium, seduced some of the natives into the habit of smoking it. From these the mania for it rapidly spread throughout the whole nation, so that in process of time the natives became feeble and enervated, submitted to foreign rule, and were completely subjugated."

We are informed by our author that "more than 50,000 chests are now annually shipped to China, taking off in return thirty-five millions of dollars, a sum greater by half than is paid on the whole imports from all other countries. According to the most recent intelligence it is estimated that the sale will reach 50,000 chests the present year." During the year 1848–9, the clear profits of the British government in India amounted to $15,488,000! And what return does this immense outlay bring to China? Nothing but loss of health, waste of property, mental imbecility, moral degradation and loss of life, evils that cannot be comprised in dollars and cents: figures and language fail to portray their magnitude and extent. Talk of the horrors of the slave trade, bad as it is, it is mercy compared to this; the horrors of the opium trade beggar description, and are worse to its victim than any outward slavery. We wish that time and space allowed us to dwell upon this theme, but we are constrained to leave it with the expression of our thanks to the author as a poor return for the obligation under which he has placed us, a sentiment in which we feel sure all will join us who may be so fortunate as to obtain a like opportunity.

